# Identification of necroptosis-related genes for predicting prognosis and exploring immune infiltration landscape in colon adenocarcinoma

**DOI:** 10.3389/fonc.2022.941156

**Published:** 2022-11-24

**Authors:** Ye Wang, Ming-gui Lin, Lei Meng, Zhang-ming Chen, Zhi-jian Wei, Song-cheng Ying, Aman Xu

**Affiliations:** ^1^ Department of General Surgery, First Affiliated Hospital of Anhui Medical University, Hefei, China; ^2^ Department of Immunology, School of Basic Medical Sciences, Anhui Medical University, Hefei, China; ^3^ Department of General Surgery, Fourth Affiliated Hospital of Anhui Medical University, Hefei, China

**Keywords:** TCGA, necroptosis, colon adenocarcinoma, prognosis model, immune

## Abstract

**Background:**

Necroptosis is a recently discovered form of cell death that plays an important role in the occurrence and development of colon adenocarcinoma (COAD). Our study aimed to construct a risk score model to predict the prognosis of patients with COAD based on necroptosis-related genes.

**Methods:**

The gene expression data of COAD and normal colon samples were obtained from the Cancer Genome Atlas (TCGA) and Genotype-Tissue Expression (GTEx). The least absolute shrinkage and selection operator (LASSO) Cox regression analysis was used to calculate the risk score based on prognostic necroptosis-related differentially expressed genes (DEGs). Based on the risk score, patients were classified into high- and low-risk groups. Then, nomogram models were built based on the risk score and clinicopathological features. Otherwise, the model was verified in the Gene Expression Omnibus (GEO) database. Additionally, the tumor microenvironment (TME) and the level of immune infiltration were evaluated by “ESTIMATE” and single-sample gene set enrichment analysis (ssGSEA). Functional enrichment analysis was carried out to explore the potential mechanism of necroptosis in COAD. Finally, the effect of necroptosis on colon cancer cells was explored through CCK8 and transwell assays. The expression of necroptosis-related genes in colon tissues and cells treated with necroptotic inducers (TNFα) and inhibitors (NEC-1) was evaluated by quantitative real-time polymerase chain reaction (qRT-PCR).

**Results:**

The risk score was an independent prognostic risk factor in COAD. The predictive value of the nomogram based on the risk score and clinicopathological features was superior to TNM staging. The effectiveness of the model was well validated in GSE152430. Immune and stromal scores were significantly elevated in the high-risk group. Moreover, necroptosis may influence the prognosis of COAD *via* influencing the cancer immune response. In *in-vitro* experiments, the inhibition of necroptosis can promote proliferation and invasion ability. Finally, the differential expression of necroptosis-related genes in 16 paired colon tissues and colon cancer cells was found.

**Conclusion:**

A novel necroptosis-related gene signature for forecasting the prognosis of COAD has been constructed, which possesses favorable predictive ability and offers ideas for the necroptosis-associated development of COAD.

## Background

Colon adenocarcinoma (COAD) is characterized by high mortality and morbidity. Despite the development in the early diagnosis and treatment of COAD, it still accounts for 880,000 estimated deaths and over 1.85 million new cases per year (1, 2). Currently, the American Joint Committee on Cancer (AJCC) TNM staging system is the main prognostic method for COAD patients (3). However, significant differences in the survival time of COAD patients with the same clinicopathologic characteristics still exist due to tumor heterogeneity (3, 4). Thus, searching for a model to predict the prognosis of COAD patients precisely is an urgent need.

Traditionally, apoptosis has been considered the only form of programmed cell death, while necrosis has been considered an accidental death that is not controlled by molecular events (5, 6). However, this concept has recently been updated, given that partial necrotic cell death has been demonstrated to be regulated by various molecular pathways. Necroptosis has been reported as a novel programmed form of cell death, whose mechanism is similar to that of apoptosis and whose form is similar to that of necrosis (7). Necroptosis can be regulated by some key molecules such as RIPK1, RIPK3, and MLKL and can also be triggered by various death receptors such as TNF receptor and Toll-like receptors (7, 8). It has been shown that necroptosis plays a key role in the regulation of cancer progression including oncogenesis and cancer immunity (6, 7, 9). However, the role of necroptosis in cancer is diverse. On the one hand, some key mediators of necroptosis-related genes were downregulated in tumor cells, suggesting that tumor cells may defend themselves from necroptosis to continue their growth. The upregulation of the expression of some regulatory factors can induce an adaptive immune response, which may prevent tumor progression. On the other hand, in some cases, necroptosis may contribute to an immunosuppressive tumor microenvironment (TME) and promote oncogenesis and cancer metastasis (9, 10). In previous studies about COAD and necroptosis, the tumor-suppressing effects of RIPK3 and RIPK1 have been studied in COAD (11). But PIPK3 has the potential to promote COAD progression by promoting tumor cell proliferation and immunosuppression (12). Therefore, it is necessary to systematically analyze the relationship between necroptosis and COAD progression.

In this study, we explored the prognostic value of necroptosis-related genes in COAD patients and built a novel nomogram model to predict the prognosis of patients with COAD. Moreover, we analyzed the correlation between necroptosis-related genes and cancer immunity and explored the potential biological mechanisms by which necroptosis-related genes influence COAD progression. Finally, the effect of necroptosis on colon cancer cells and the expression of necroptosis-related genes in cells and tissues were evaluated.

## Materials and methods

### Data acquisition

The gene expression matrix of normal colon tissues was downloaded from The Cancer Genome Atlas (TCGA) and the Genotype-Tissue Expression (GTEx) databases. The TCGA and GTEx databases contained 41 normal colon samples and 308 normal colon samples, respectively. The gene expression matrix of the colon cancer samples and their clinical information were acquired from the TCGA database, which contained 473 COAD samples. Samples lacking complete clinical data or with an overall survival (OS) of 0 days were excluded. Finally, we included 425 COAD samples from the TCGA in the follow-up work. The GSE152430 dataset, containing 48 COAD samples with complete clinical information, was downloaded from the Gene Expression Omnibus (GEO) database to further validate the reliability of the analysis. The gene expression data from the TCGA and GEO were both normalized by log2 (transcripts per kilobase million (TPM) + 0.01). We used the GeneCards database (https://www.genecards.org/) to screen out necroptosis-related genes (13). These genes are shown in **Supplementary Data Sheet S1**.

### Bioinformation analysis

The Wilcoxon test was used to identify necroptosis-related differentially expressed genes (DEGs) between normal colon samples and COAD samples from the TCGA cohort with a false discovery rate (FDR) <0.05. Univariate Cox analysis was utilized to select prognostic necroptosis-related genes in the TCGA cohort. The common genes between DEGs and prognostic necroptosis-related genes were selected as the candidate genes. In addition, the correlation among the candidate genes was analyzed by Spearman correlation analysis in the TCGA cohort. Metascape (http://metascape.org) is a reliable, intuitive tool for gene annotation and gene list enrichment analysis. Based on the functional annotation of gene/protein lists, Metascape can facilitate data-driven decisions (14). In this study, Metascape was used to conduct pathway and process enrichment analysis of the candidate genes.

Furthermore, the candidate genes were then included in the least absolute shrinkage and selection operator (LASSO) regression analysis to construct a prognostic model and determine the LASSO genes to minimize the level of overfitting *via* “glmnet” (version 4.1.1) R package in the TCGA cohort (15). To reduce the potential instability of the results, a three-fold cross-validation was conducted and the optimal tuning parameter *λ* was identified according to a 1-SE (standard error) standard. The risk score was calculated based on the corresponding regression coefficients and the expression of each gene as follows: risk score = (gene expression level × corresponding coefficient). The optimal cutoff value was determined based on the log-rank statistic by the “surv_cutpoint” function of the package “survminer” (version 0.4.9) (16). Patients with COAD in the TCGA cohort were separated into high- and low-risk groups based on the optimal cutoff value. The expression levels of LASSO genes between the low-risk group and the high-risk group were compared by the Wilcoxon test. Otherwise, the Human Protein Atlas (HPA) was used to analyze the expression of proteins encoded by the LASSO genes. OS and progression-free survival (PFS) were calculated to analyze survival differences between the high- and low-risk groups in the TCGA cohort by R package “survival” (version 3.1.10). Univariate and multivariate Cox regression analyses for OS and PFS were carried out to identify independent prognostic factors of COAD in the TCGA cohort.

Based on the results of the multivariate Cox regression analysis, we constructed nomogram models to help us make a better clinical prediction of OS and PFS for COAD patients in the TCGA cohort by using the “survival” (version 3.1.10) and “rms” (version 6.2.0) packages (17). Moreover, the calibration curve showed the difference between the predicted results of the nomogram and the actual results in the TCGA cohort. Decision curve analysis (DCA) was utilized to assess the effectivity of the constructed nomogram compared with the TNM stage (18).

The GSE152430 dataset as a validation cohort was used to validate the repeatability of the risk model. The risk score was calculated based on the formula of the training cohort. Patients with COAD in the validation cohort were separated into high- and low-risk groups based on the optimal cutoff value of the training cohort. The survival analysis was used to analyze differences in OS and PFS between the high-risk and low-risk groups in the validation cohort. The calibration curve was used to assess the accuracy of the predicted results of the nomogram in the validation cohort. In addition, the expression levels of LASSO genes between the low-risk group and the high-risk group were compared by the Wilcoxon test in the validation cohort.

To investigate the cancer immunity of COAD patients, the stromal/immune scores were determined *via* the “ESTIMATE” (version 1.0.13) package (19). According to the expression levels of immune cell-specific markers, the level of immune infiltration was determined by single-sample gene set enrichment (ssGSEA) with the R package “gsva” (version 1.38.2), which was recorded as the ssGSEA score (20). Finally, TME and 16 immune cell types were evaluated in the different risk groups.

DEGs between the two risk groups with *P <*0.05 and log2 (fold change) >1 or <−1 were selected. DEGs were included in the functional enrichment analysis. The Gene Ontology (GO) analysis of biological processes (BP), cellular components (CC), and molecular functions (MF) and WikiPathways analysis were performed by Metascape, and terms with *P*-values <0.01 were regarded as significant (21).

**Figure 1:**
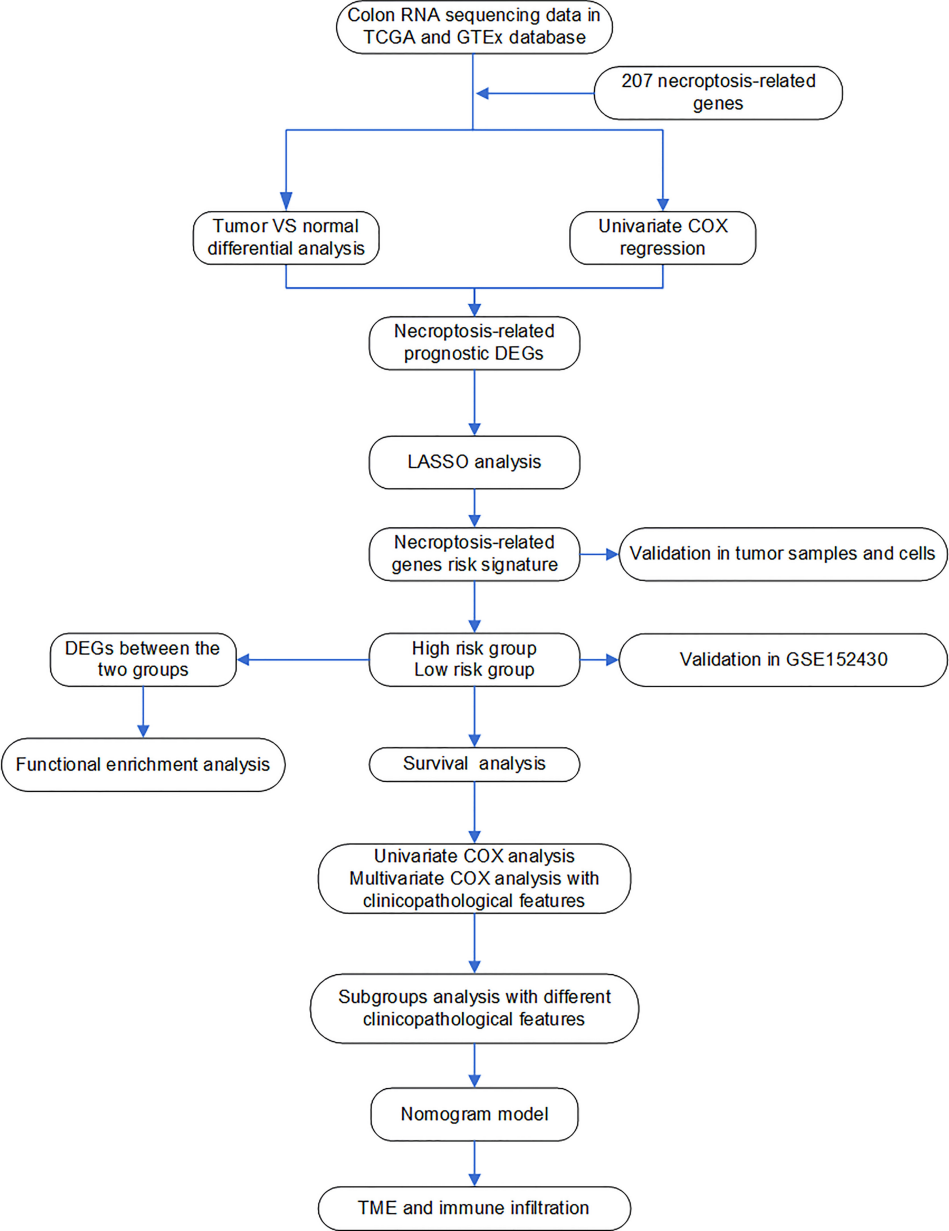
Flow chart of the analyses used in this study.

All processes of our study are shown in **Figure 1**.

### Cell culture and administration of drugs

The HCT116 and SW480 human colon cancer cell lines (Stem Cell Bank, Chinese Academy of Sciences, Shanghai, China) were cultured in high glucose Dulbecco’s modified Eagle’s medium (DMEM) (Gibco; Thermo Fisher Scientific, Inc., Waltham, MA, USA) supplemented with 10% fetal bovine serum (FBS; Gibco; Thermo Fisher Scientific, Inc., Waltham, MA, USA) in 5% CO_2_ at 37°C. A stock solution of necrostatin-1 (NEC-1; Apexbio, Houston, USA) was prepared in DMSO at a concentration of 20 mM, and the working solution was diluted into 0.1% with DMEM. TNFα (PeproTech, Suzhou, China) was dissolved in stock solution at a concentration of 100 μg/ml and diluted to 100 ng/ml for actual use. Some cells were pretreated with NEC-1 for 6 h. Subsequently, TNFα was added to the cells in the absence or presence of NEC-1 for 24 h prior to cell collection.

### Cell viability assay

The cell counting kit-8 (CCK-8; Beyotime, Shanghai, China) was used to monitor cell proliferation. Briefly, the cells were seeded in 96-well plates in 100 μl of culture medium without or with NEC-1 and TNFα, and then incubated in a 37°C, 5% CO_2_ incubator. After culturing for 0, 12, or 24 h, 10 μl of the CCK-8 reagent was added to each well, and the cells were incubated for 2 h at 37°C. Finally, the optical density was measured at 450 nm using a universal microplate reader (BioTek, Winooski, USA).

### Migration and invasion assays

Migration and invasion assays were performed in 24-well cell culture chambers (Costar 3422; Corning Inc., Corning, NY, USA), and the lower and upper chambers were separated by a polycarbonate membrane (8-μm pore size). About 4 × 10^4^ cells stimulated without or with NEC-1 and TNFα were seeded on the upper chamber with DMEM without FBS. Moreover, the upper chambers were precoated with Matrigel (BD, Franklin Lakes, USA) for the invasion assay rather than for the migration assay. DMEM containing 10% FBS was added to the lower chamber. After incubation for 24–48 h at 37°C with 5% CO_2_, we removed the cells remaining on the upper membrane with cotton wool. At the same time, the cells on the other side of the membrane were fixed with methanol and stained with 0.1% crystal violet solution. Finally, a microscope (Olympus, Tokyo, Japan) was used to observe the cells.

### Western blotting

All protein samples from the cells were extracted using RIPA reagent containing 100 μg/ml of PMSF (Beyotime, Shanghai, China) and boiled to denaturation for 5 min. The concentrations of the protein samples were evaluated with the bicinchoninic acid (BCA) protein assay kit (Beyotime, Shanghai, China). The protein samples were isolated by 10% SDS–PAGE (Beyotime, Shanghai, China) and later transferred to polyvinylidene fluoride (PVDF) membranes (Merck Millipore, Shanghai, China). The membranes were blocked with TBS with 0.1% Tween (TBST) containing 5% skim milk for 2 h and then incubated at 4°C overnight with the following antibodies: anti-RIPK1 (R25595, 1:1000; ZENBIO, Chengdu, China), anti-P-RIPK1 (66854-1-Ig, 1:1,000; Proteintech, Wuhan, China), anti-P-RIPK3 (ab209384, 1:1,000; Abcam, Cambridge, UK), anti-P-MLKL (382136, 1:1,000; ZenBio, Chengdu, China), and anti-GAPDH (60004-1-Ig, 1:1,000; Proteintech, Wuhan, China). After washing three times with TBST, the membranes were incubated with the secondary antibody for 1.5 h at room temperature and later visualized by enhanced chemiluminescence (ECL) (Thermo Fisher Scientific, Waltham, MA, USA). The qualification of Western blotting was performed using ImageJ.

### Extraction of RNA and quantitative real-time polymerase chain reaction

The tissue specimens of 16 COAD patients were collected from the Department of General Surgery, First Affiliated Hospital of Anhui Medical University. Experiments using patients’ specimens were approved by the Institutional Ethics Committee, First Affiliated Hospital of Anhui Medical University. Total RNAs were extracted from colon cancer tissues and cells using a total RNA Quick Extraction Kit (Generay Biotech, Shanghai, China) in accordance with the manual. Then, the extracted RNAs were reverse-transcribed into cDNAs using the PrimeScript™ RT Master Mix (TaKaRa, Dalian, Liaoning, China). qRT-PCR was performed with TB Green™ Premix Ex Taq™ II (Takara, Dalian, Liaoning, China) in ABI Prism 7900HT/FAST (Applied Biosystems, USA). The relative expression levels were analyzed *via* the 2^−ΔΔCT^ method normalized by GAPDH expression. The Wilcoxon test was used to analyze the differences in gene expression between the cancer and normal samples. *t*-test was used to evaluate the differences in gene expression between cells treated without or with NEC-1 and TNFα. The primer sequences used in our study are shown in **Supplementary Data Sheet S2**.

### Statistical analysis

The statistical analysis and plots were performed in R version 4.0.5 (https://cran.r-project.org/bin/windows/base) and GraphPad Prism 7.0. All categorical variables were tested by the chi-square test. The independent prognostic factors for COAD were identified by univariate and multivariate Cox analyses. *P-*values <0.05 were considered significant.

## Results

### Identification of prognostic necroptosis-related genes in the COAD samples

**Figure 2:**
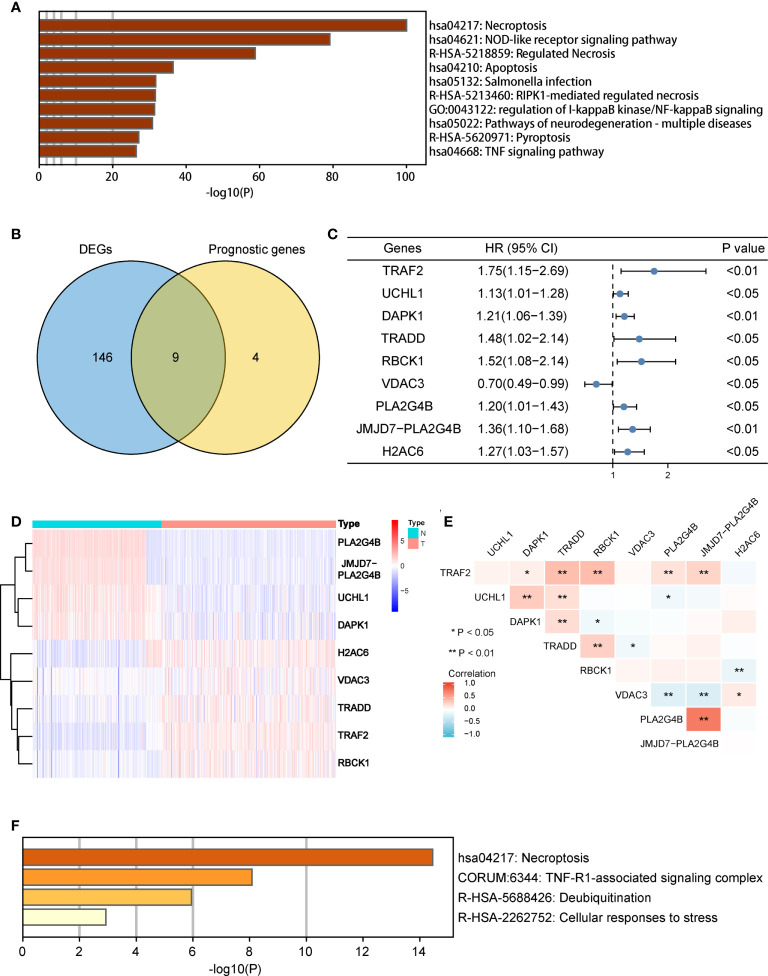
Identification of candidate necroptosis-related DEGs in TCGA cohort. **(A)** Function enrichment analysis of 155 DEGs by Metascape. **(B)** Venn diagram to identify candidate genes by taking the intersection between the prognostic genes and DEGs. **(C)** Forest plot showing the results of univariate Cox regression analysis of nine candidate genes. **(D)** Heat map showing the expression of nine candidate genes in normal tissues and COAD tissues. **(E)** Spearman’s correlation analysis of the nine candidate genes. **(F)** Function enrichment analysis of nine candidate genes by Metascape. DEGs, differentially expressed genes; OS, overall survival; TCGA, the Cancer Genome Atlas. *P < 0.05, **P < 0.01.

There were 155 necroptosis-related DEGs (FDR < 0.05) between adjacent normal and tumor tissues were identified. To analyze the potential biological functions of these DEGs, we conducted function enrichment analysis using Metascape. As we expected, the results revealed that the most significant enriched terms were involved in necroptosis (**Figure 2A**). Then 13 necroptosis-related genes associated with OS in tumor tissues were identified. Finally, 9 common genes were selected as candidate genes for further study (**Figure 2B**). The prognosis and expression of the candidate genes in COAD samples are shown in **Figures 2C, D**. And the results of the correlation analysis among the candidate genes are shown in **Figure 2E**. Finally, the candidate genes were also enriched in the process of necroptosis (**Figure 2F**).

### Construction of a necroptosis-related gene prognostic risk model

**Figure 3:**
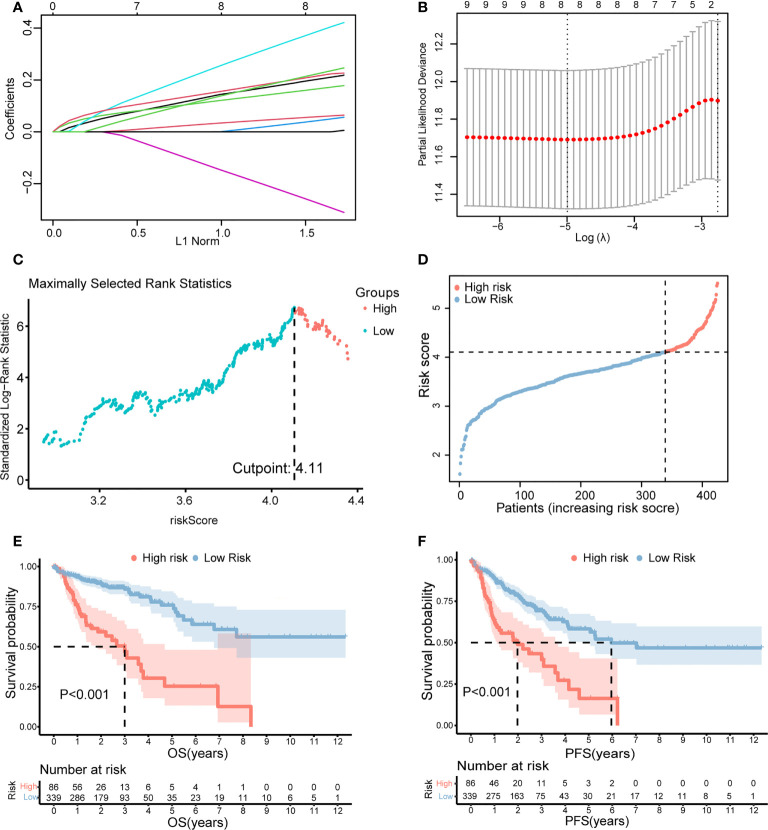
Establishment of the risk score by LASSO regression analysis. **(A)** LASSO coefficient profiles of the eight genes in colon cancer samples. **(B)** A coefficient profile plot was generated against the log (lambda) sequence. Selection of the optimal parameter (lambda) in the LASSO model for colon cancer. **(C)** The optimal cutoff value was determined to maximize log-rank statistic. **(D)** The distribution and cutoff value of the risk scores in the TCGA cohort. Kaplan–Meier curves for the OS **(E)** and PFS **(F)** of patients in the high- and low-risk groups in the TCGA cohort. LASSO, least absolute shrinkage and selection operator; TCGA, The Cancer Genome Atlas; OS, overall survival; PFS, progression-free survival.

**Figure 4:**
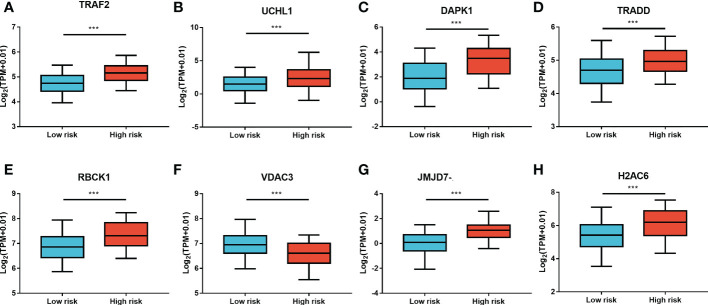
The expression levels of LASSO genes between the low-risk group and the high-risk group. The expression of the eight necroptosis-related genes [*TRAF2* **(A)**, *UCHL1* **(B)**, *DAPK1* **(C)**, *TRADD* **(D)**, *RBCK1* **(E)**, *VDAC3* **(F)**, *JMJD7-PLA2G4B* **(G)**, *H2AC6* **(H)**] between the low-risk group and the high-risk group in the TCGA cohort. **P < 0.01, ***P < 0.001.

Furthermore, LASSO regression analysis was used to construct a necroptosis-related gene signature based on nine candidate genes. Finally, eight genes as LASSO genes were determined given the optimal value of *λ*, and a risk score formula was calculated (**Figures 3A, B**) as follows: risk score = (*TRAF2* × 0.20 + *UCHL1* × 0.057 + *DAPK1* × 0.164 + *TRADD* × 0.041 + *RBCK1* × 0.381 − *VDAC3* × 0.269 + *JMJD7-PLA2G4B* × 0.213 + *H2AC6* × 0.220). Then, the optimal cutoff value of the risk score was determined to be 4.11 (**Figure 3C**). The patients were split into low- and high-risk groups based on the optimal cutoff value (**Figure 3D**). Compared with the OS and PFS of the low-risk group, the OS and PFS of patients in the high-risk group were shorter significantly (**Figures 3E, F**). In addition, the expression levels of LASSO genes were different significantly between the low-risk group and the high-risk group (**Figures 4A–H**) and the expression levels of classical executors of necroptosis (RIPK1, RIPK3, MLKL, TNF) in high and low risk groups in the TCGA were showed in **Supplementary Image S1(A)**.

### Prognostic value of the risk score model

**Figure 5:**
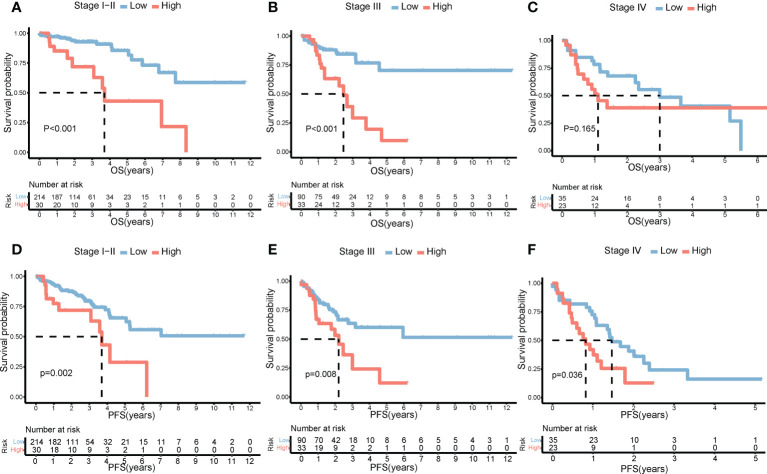
Impact of risk score on prognosis by subgroup analysis. OS differences between the high- and low-risk groups in stages I–II **(A)**, stage III **(B)**, and stage IV **(C)**. PFS differences between the high- and low-risk groups in stages I–II **(D)**, stage III **(E)**, and stage IV **(F)**. OS, overall survival; PFS, progression-free survival.

**Figure 6:**
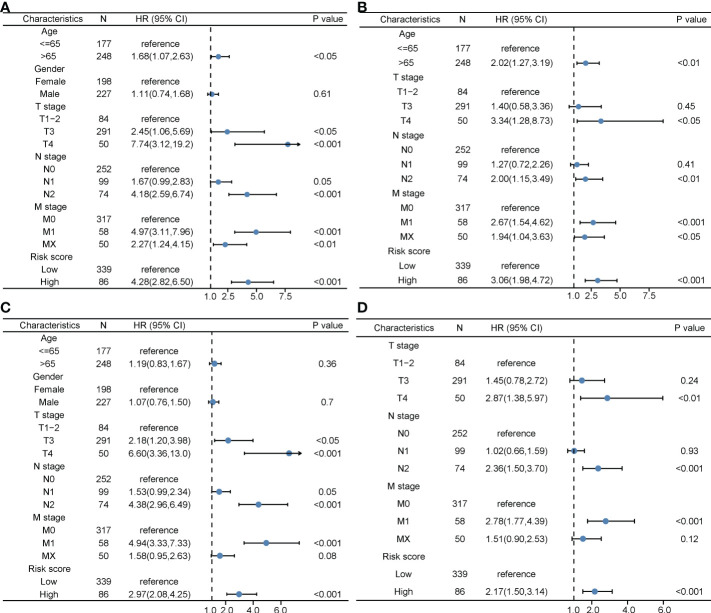
The prognostic value of clinical features and the risk score. **(A)** Univariate Cox regression analyses regarding OS in the TCGA cohort. **(B)** Multivariate Cox regression analyses regarding OS in the TCGA cohort. **(C)** Univariate Cox regression analyses regarding PFS in the TCGA cohort. **(D)** Multivariate Cox regression analyses regarding PFS in the TCGA cohort. OS, overall survival; TCGA, The Cancer Genome Atlas; PFS, progression-free survival.

The clinical characteristics between the high-risk group and the low-risk group in the TCGA cohort are shown in **Table 1**. The T stage, N stage, and M stage in the high-risk group were more advanced than those in the low-risk group. Subgroup survival analysis showed the OS and PFS of the high- and low-risk groups in stages I–II, stage III, and stage IV in the TCGA cohort (**Figures 5A–F**). After univariate and multivariate Cox regression analyses, the T group, N group, M group, age group, and risk score were selected as independent risk factors for OS and PFS of COAD patients in the TCGA cohort (**Figures 6A–D**). Among these factors, the risk score was significantly related to OS [univariate: hazard ratio (HR), 4.28; 95% CI, 2.82–6.50; *P* < 0.001; multivariate: HR, 3.06; 95% CI, 1.98–4.72; *P* < 0.001] and PFS (univariate: HR, 2.97; 95% CI, 2.08–4.25; *P* < 0.001; multivariate: HR, 2.17; 95% CI, 1.50–3.14; *P* < 0.001).

**Table 1:** Clinical characteristics of the colon patients between the high- and low-risk groups.

Variables	Training data (*n* = 425)		*P*-value	Validation data (*n* = 48)		*P*-value
	Low risk	High risk		Low risk	High risk	
Age			0.867			0.435
≤65	140 (41.3%)	37 (43.0%)		5 (23.8%)	3 (11.1%)	
>65	199 (58.7%)	49 (57.0%)		16 (76.2%)	24 (88.9%)	
Gender			0.535			NA
Female	161 (47.5%)	37 (43.0%)		–	–	
Male	178 (52.5%)	49 (57.0%)		–	–	
T stage			0.014			0.430
T1–T2	73 (21.5%)	11 (12.8%)		–	–	
T3	233 (68.7%)	58 (67.4%)		18 (85.7%)	26 (96.3%)	
T4	33 (9.7%)	17 (19.8%)		3 (14.3%)	1 (3.7%)	
N stage			<0.001			NA
N0	218 (64.3%)	34 (39.5%)		21 (100.0%)	27 (100.0%)	
N1	74 (21.8%)	25 (29.1%)		–	–	
N2	47 (13.9%)	27 (31.4%)		–	–	
M stage			<0.001			NA
M0	266 (78.5%)	51 (59.3%)		21 (100.0%)	27 (100.0%)	
M1	35 (10.3%)	23 (26.7%)		–	–	
MX	38 (11.2%)	12 (14.0%)		–	–	
Stage			<0.001			NA
Stage I	66 (19.5%)	9 (10.5%)		–	–	
Stage II	148 (43.7%)	21 (24.4%)		21 (100.0%)	27 (100.0%)	
Stage III	90 (26.5%)	33 (38.4%)		–	–	
Stage IV	35 (10.3%)	23 (26.7%)		–	–	

NA, not available.

### The nomogram model for COAD patients

**Figure 7:**
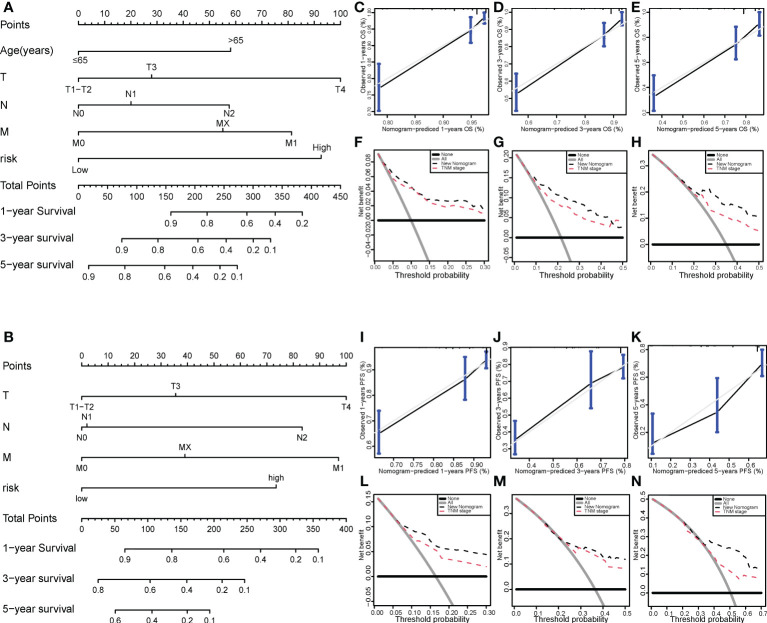
Nomogram model for colon cancer in the TCGA cohort. Nomogram models to predict the 1-, 3-, and 5-year OS rates **(A)** and PFS rates **(B)** of colon cancer cases. Calibration graphs indicate that the predicted 1- **(C)**, 3- **(D)**, and 5-year **(E)** OS rates correspond with the actual OS rates. DCA showing the net benefit of our prognosis model and the TNM staging model in the predictive models of 1- **(F)**, 3- **(G)**, and 5-year OS rates **(H)**. Calibration graphs indicate that the predicted 1- **(I)**, 3- **(J)**, and 5-year **(K)** PFS rates correspond with the actual PFS rates. DCA showing the net benefit of our prognosis model and the TNM staging model in the predictive model of 1- **(L)**, 3- **(M)**, and 5-year PFS rates **(N)**. TCGA, The Cancer Genome Atlas; DCA, decision curve analysis; OS, overall survival; PFS, progression-free survival.

We then successfully built nomogram models to predict the OS and PFS of COAD patients according to the multivariate model (**Figures 7A, B**). The C-index value of the nomogram for OS was 0.784 and the C-index value of the nomogram for PFS was 0.73. As shown in **Figures 7C–E**, the predicted 1-, 3-, and 5-year OS probabilities were similar to the actual observations. Moreover, the predicted 1-, 3-, and 5-year PFS probabilities were also similar to the actual observations (**Figures 7F–H**). Compared with the TNM stage model, DCA demonstrated that our prognosis model to predict the OS and PFS of COAD patients was more effective (**Figures 7I–N**).

### Validation of the risk score model

**Figure 8:**
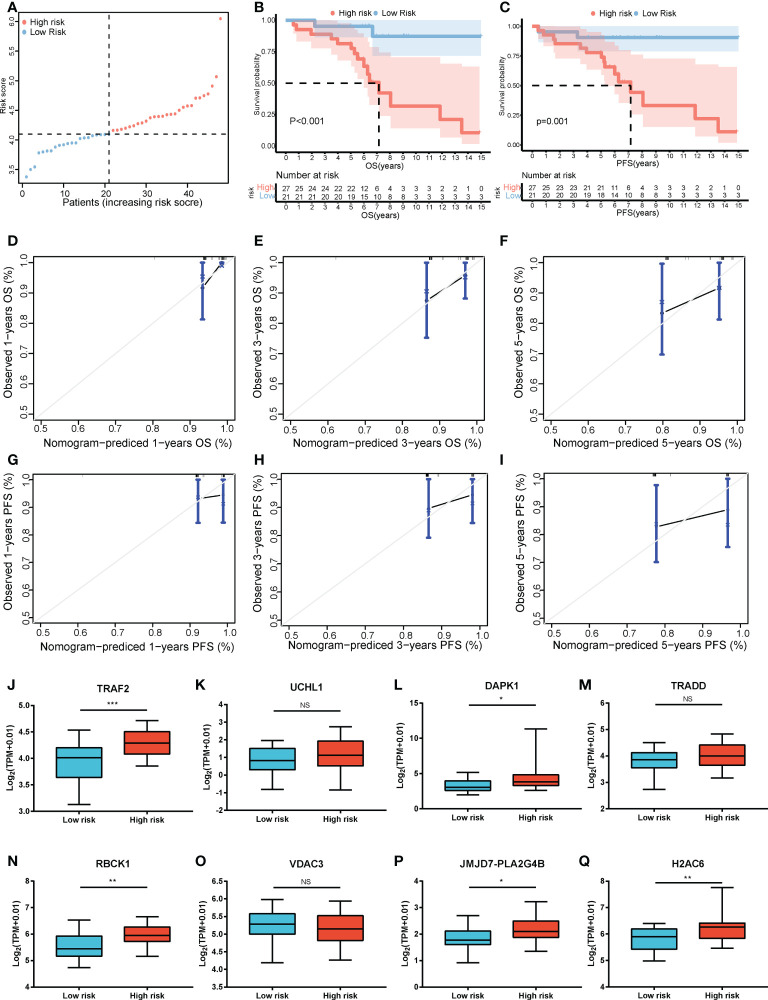
Validation of the risk score model. **(A)** The distribution and cutoff value of the risk scores in the GSE152430 cohort. Kaplan–Meier curves for the OS **(B)** and PFS **(C)** of patients in the high- and low-risk groups in the GSE152430 cohort. Calibration graphs indicate that the predicted 1- **(D)**, 3- **(E)**, and 5-year **(F)** OS rates correspond with the actual survival rates. Calibration graphs indicate that the predicted 1- **(G)**, 3- **(H)**, and 5-year **(I)** PFS rates correspond with the actual survival rates. The expression of the eight necroptosis-related genes [*TRAF2* **(J)**, *UCHL1* **(K)**, *DAPK1* **(L)**, *TRADD* **(M)**, *RBCK1* **(N)**, *VDAC3* **(O)**, *JMJD7-PLA2G4B* **(P)**, *H2AC6* **(Q)**] between the low-risk group and the high-risk group in the GSE152430 cohort. *P < 0.05, **P < 0.01, ***P < 0.001. OS, overall survival; PFS, progression-free survival. NS, no significance.

The risk score formula of the training cohort was further used in the validation cohort to verify the prognostic significance of the constructed risk score model. The patients in the validation cohort were divided into low- and high-risk groups based on the same optimal cutoff value of 4.11 (**Figure 8A**). The OS and PFS of patients in the high-risk group were shorter (**Figures 8B, C**). The predicted 1-, 3-, and 5-year OS and PFS probabilities were similar to the actual observations in the validation cohort, which further verified the robustness of the predictive model (**Figures 8D–I**). In addition, the expression levels of the LASSO genes between the high-risk group and the low-risk group are shown in **Figures 8J–Q** and the expression levels of RIPK1, RIPK3, MLKL, TNF in high and low risk groups in the validation cohort were showed in **Supplementary Image S1(B)**. The clinical characteristics between the high-risk group and the low-risk group in the validation cohort are also shown in **Table 1**.

### Cancer immunity analysis and functional enrichment analyses in the two risk groups

**Figure 9:**
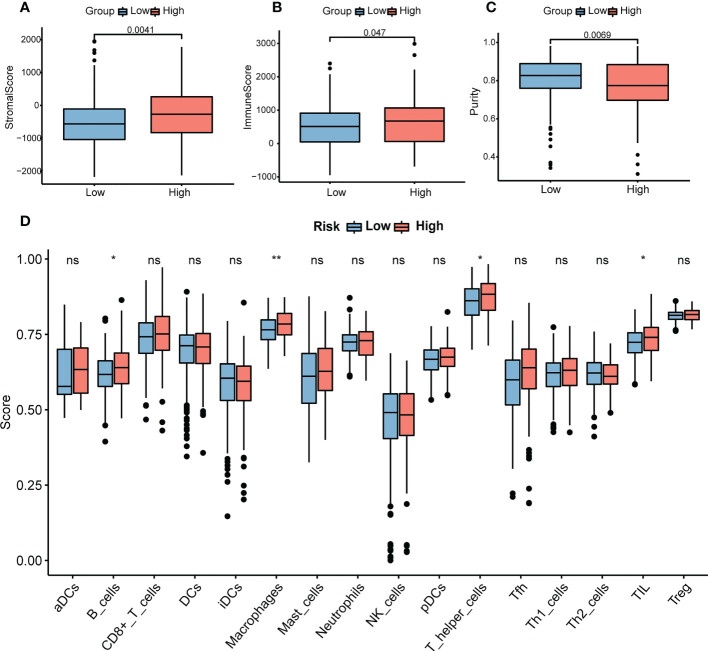
Analysis of immune infiltration in COAD. **(A)** The stromal scores in the high- and low-risk groups. **(B)** The immune scores in the high- and low-risk groups. **(C)** The purity scores in the high- and low-risk groups. **(D)** The content of 16 immune cells between the high- and low-risk groups. *P*-values were obtained using *t*-test. *P < 0.05, **P < 0.01. COAD, colon adenocarcinoma. NS, no significance.

**Figure 10:**
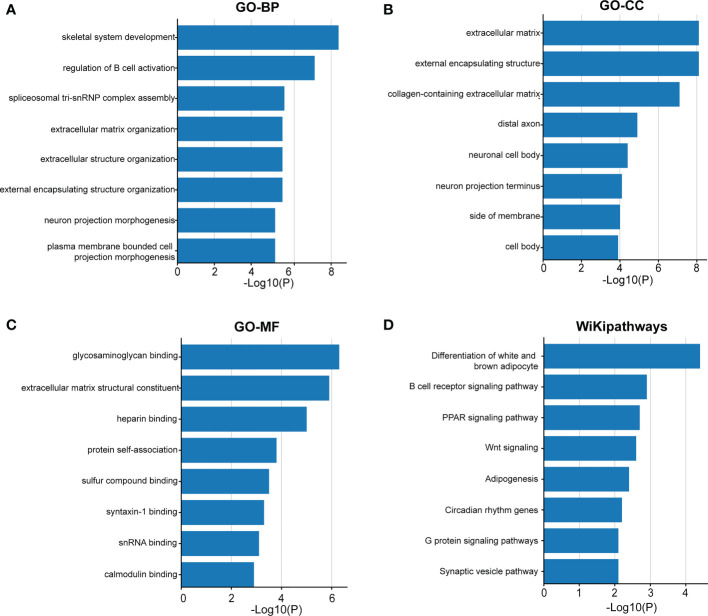
The functional enrichment analysis of the DEGs between the two groups. Gene Ontology analysis of BP **(A)**, CC **(B)**, MF **(C)** and WikiPathways **(D)** analysis of the DEGs between the high- and low-risk groups. BP, biological processes; CC, cellular components; MF, molecular functions; DEGs, differentially expressed genes.

Immune and stromal scores were significantly higher, and tumor purity was lower in the high-risk group than in the low-risk group (**Figures 9A–C**). Moreover, the high-risk group contained more B cells, macrophages, and T helper cells (*P* < 0.05) (**Figure 9D**). We also analyzed the enrichment information of DEGs between the two risk groups. The GO (BP, CC, MF) and WikiPathways analyses showed that DEGs were mainly enriched in immune-related processes (**Figures 10A–D**). The detailed results of the functional enrichment analyses are shown in **Supplementary Data Sheet S3**.

### Validation of the expression of the necroptosis-related genes in colon cancer tissues and cells

**Figure 11:**
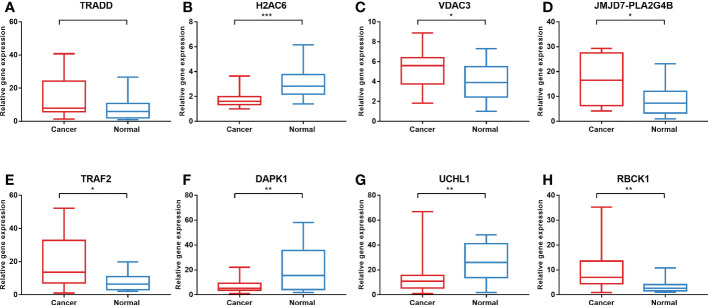
Validation of gene expression in tissues *via* qRT-PCR. The expression of eight necroptosis-related genes [*TRADD* **(A)**, *H2AC6* **(B)**, *VDAC3* **(C)**, *JMJD7-PLA2G4B* **(D)**, *TRAF2* **(E)**, *DAPK1* **(F)**, *UCHL1* **(G)**, *RBCK1* **(H)**] in the normal and cancer tissues. *P < 0.05, **P < 0.01, ***P < 0.001. qRT-PCR, quantitative real-time polymerase chain reaction.

To validate the expression levels of the necroptosis-related genes from the prognostic signature, we first detected the expression levels of eight necroptosis-related genes in 16 colon cancer tissues and adjacent normal mucosa. The clinical characteristics of 16 patients are shown in **Supplementary Table S1**. Our results showed that *TRAF2*, *RBCK1*, *JMJD7-PLA2G4B*, and *VDAC3* were upregulated, while *UCHL1*, *DAPK1*, and *H2AC6* were downregulated significantly in the COAD samples compared with those in normal colon samples. The expression levels of eight necroptosis-related genes in human colon cancer and normal colon samples are shown in **Figure 11**.

**Figure 12:**
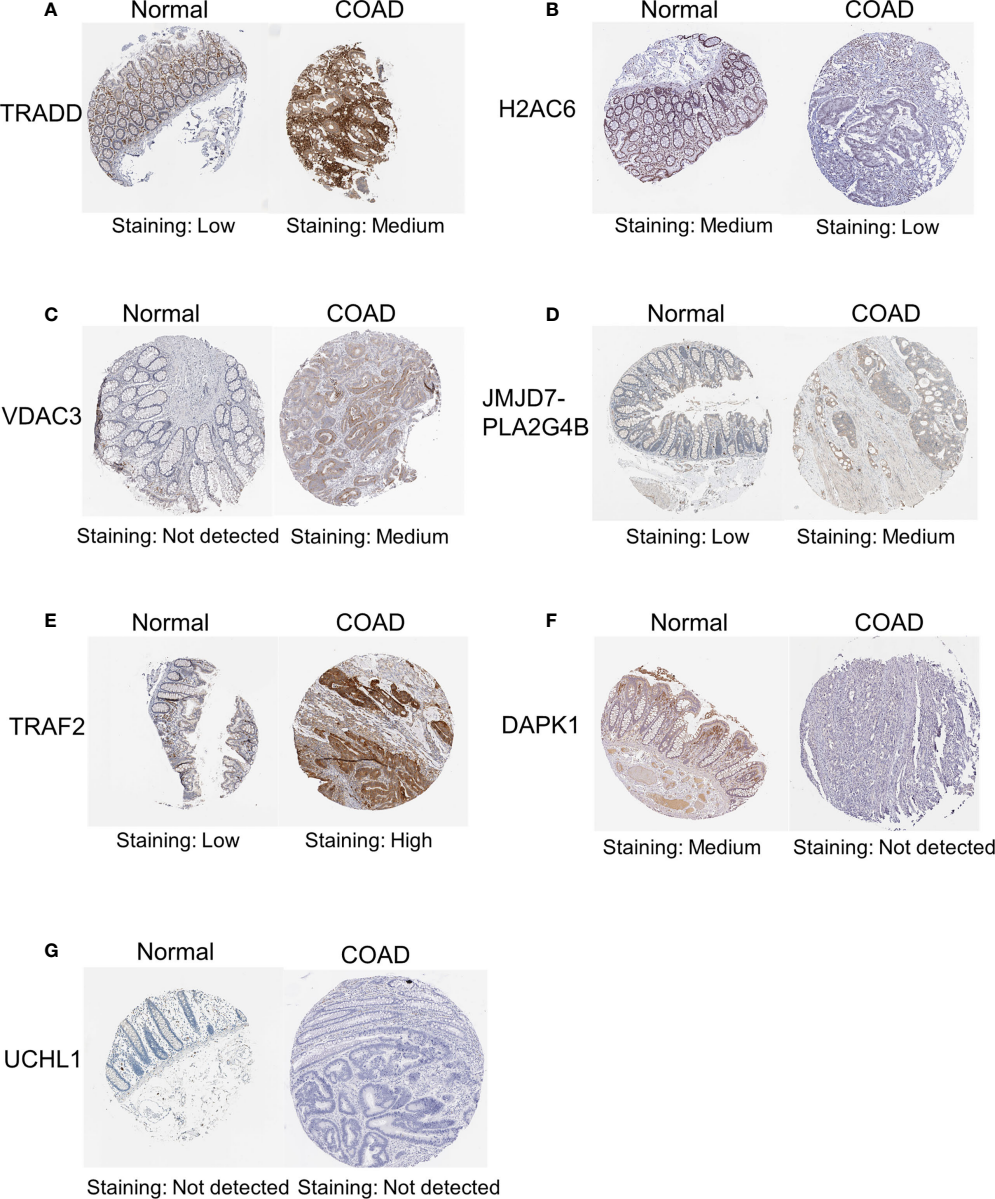
IHC analysis of the protein expression of necroptosis-related candidate genes in COAD and normal tissues in the HPA database. The protein expression levels of *TRADD* **(A)**, *H2AC6* **(B)**, *VDAC3* **(C)**, *JMJD7-PLA2G4B* **(D)**, TRAF2 **(E)**, *DAPK1* **(F)** and *UCHL1* **(G)** in colon cancer tissues and normal tissues in the HPA database. IHC, immunohistochemical.

In addition, to further validate the protein expression levels of necroptosis-related candidate genes in COAD specimens and normal colon tissues, we performed immunohistochemical (IHC) staining analysis from the HPA database (**Figure 12**). IHC staining analysis suggested that *TRADD*, *VDAC3*, *JMJD7-PLA2G4B*, and *TRAF2* expression levels were upregulated in COAD tumor tissues compared with those in normal gastric tissues at the protein level, which was consistent with the expression pattern of the genes. Furthermore, the protein levels of *DAPK1* and *H2AC6* were downregulated in COAD samples compared with those in normal colon samples. However, the protein level of *UCHL1* was not detected in the HPA database.

**Figure 13:**
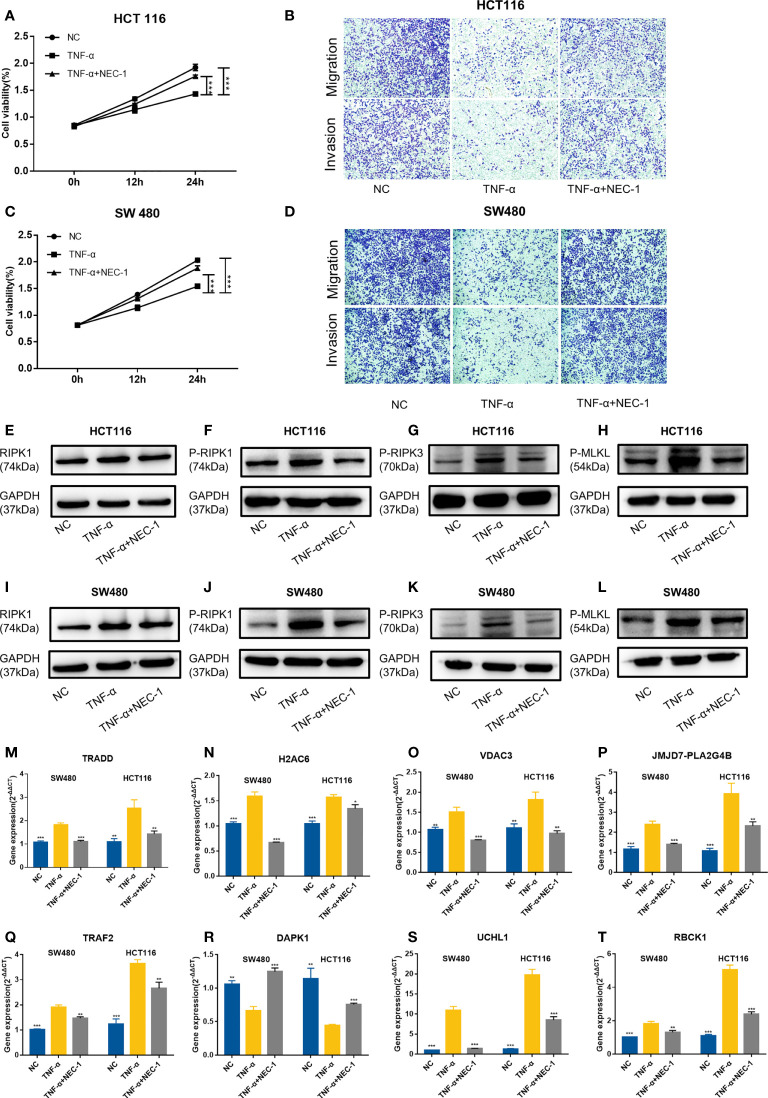
Evaluation of gene expression in the HCT116 cell and SW480 cell treated with or without TNFα/NEC-1. TNFα inhibited the viability of cells and NEC-1 can counteract the effects of TNFα on HCT116 cells **(A)** and SW480 cells **(C)**. TNFα inhibited the migration and invasion ability of cells and NEC-1 can counteract the effects of TNFα on HCT116 cells **(B)** and SW480 cells **(D)**. Representative images of the Western blot analysis of RIPK1 **(E)**, P-RIPK1 **(F)**, P-RIPK3 **(G)**, and P-MLKL **(H)** in the HCT116 cells and RIPK1 **(I)**, P-RIPK1 **(J)**, P-RIPK3 **(K)**, and P-MLKL **(L)** in the SW480 cells treated with or without TNFα/NEC-1. Eight necroptosis-related genes [*TRADD* **(M)**, *H2AC6* **(N)**, *VDAC3* **(O)**, *JMJD7-PLA2G4B* **(P)**, *TRAF2* **(Q)**, *DAPK1* **(R)**, *UCHL1* **(S)**, *RBCK1* **(T)**] in the cells treated with or without TNFα/NEC-1. *P < 0.05, **P < 0.01, ***P < 0.001 versus the TNFα group.

Then, we used TNFα to stimulate the human colon cancer cells HCT116 and SW480 and used NEC-1 to inhibit the occurrence of necroptosis in the cells. TNFα could inhibit the viability, migration, and invasion ability of the cells, while NEC-1 could counteract the effects of TNFα on the cells (**Figures 13A–D**). Western blot analysis demonstrated that the protein levels of RIPK1, P-RIPK1, P-RIPK3, and P-MLKL, which were key molecules of necroptosis, increased in the cells treated with TNFα and decreased in the cells treated with NEC-1 (**Figures 13E–L**). qPCR results demonstrated that *UCHL1*, *TRADD*, *RBCK1*, *JMJD7-PLA2G4B*, *H2AC6*, *TRAF2*, and *VDAC3* were upregulated in cells treated with TNFα and downregulated in cells treated with NEC-1, while the expression trend of *DAPK1* was the opposite (**Figures 13M–T**).

## Discussion

Recently, a growing number of studies have shown that necroptosis plays a key role in tumor progression (9, 11, 22). However, the effect of necroptosis on tumor cells is complex, and its dual impact of reducing and promoting the growth of tumor cells was shown in different types of cancer. On the one hand, the downregulation of the expression of numerous key molecules in necroptosis such as RIPK1, RIPK3, and MLKL has been found in different types of cancer cells, suggesting that cancer cells may evade necroptosis to survive (8, 9, 22, 23). On the other hand, necroptosis can accelerate tumor metastasis in some cases. For instance, the knockout of the necroptosis key molecules such as RIPK1, RIPK3, or MLKL in cancer cells markedly reduced tumorigenicity (24, 25). Moreover, the role of necroptosis in tumor immunity is also complex. RIPK3 has been found to be necessary for regulating the expression of cytokines in dendritic cells (DCs), a key security guard for regulating immune homeostasis by expressing regulatory cytokines and linking the innate and adaptive immune systems (26). Necroptosis can also activate the adaptive immune response by releasing damage-associated molecular patterns (DAMPs) into a tissue microenvironment (27). Although necroptosis plays a role in the induction of tumor immunity to limit tumor growth, there is also evidence suggesting that necroptosis-induced immune inflammatory cells can promote tumor progression by promoting angiogenesis and cancer cell proliferation and accelerating cancer metastasis (10, 25). For example, inflammatory cells activated by necroptosis also release reactive oxygen species (ROS), which can damage DNA to promote the progression of tumors (28). In COAD, RIPK3-mediated inflammation can promote intestinal tumors by inducing an immune-suppressive tumor microenvironment, and RIPK1 has been shown to interact with mitochondrial Ca2^+^ uniporter to promote colorectal oncogenesis (12, 29). Thus, in consideration of the vital role of necroptosis, we investigated the expression levels of necroptosis-related genes in COAD to explore whether these genes could serve as significant biomarkers for COAD prognosis and influence the progression and TME in COAD.

Through studying the prognostic effect and expression level of necroptosis-related genes at the RNA level, eight LASSO genes were determined to identify a prognostic model with the LASSO method, a suitable method for high-dimensional data which can prevent overfitting and obtain an interpretable prediction rule (15). The Kaplan–Meier analysis showed that the risk score group had great validity. However, in the subgroup survival analysis, there was no significant difference in the OS between the high-risk group and the low-risk group in stage IV. However, the median survival time of the high-risk group was shorter than that of the low-risk group in stage IV. This risk score was then considered an independent prognostic factor for COAD through univariate and multivariate Cox regression analyses. Currently, the AJCC TNM staging system is the main prognostic method for COAD patients in the clinical process. However, there was a significant difference in OS and PFS of COAD patients with the same clinicopathologic characteristics due to tumor heterogeneity (4). So, the nomogram was built to facilitate the prediction of the OS and PFS of COAD patients. Calibration curves showed the great predictive performance of this nomogram, and DCA curves demonstrated that the novel nomogram can serve as a great model as it outperforms the TNM stage prediction model for patients with COAD. In addition, the predictive power of our risk group model was also great in the GSE152430 dataset. These results demonstrated that our model had a certain applicability.

The prognostic model in this study was composed of eight necroptosis-related genes which were directly or indirectly involved in the process of necroptosis. In previous studies, *UCHL1* was reported to play a major role in promoting podocyte necroptosis by regulating the ubiquitination state of the RIPK1/RIPK3 pathway (30). *TRADD* acted as a partner of RIPK3 to initiate necroptosis in response to TNF stimulation (31). Histone H2A family members can act as DAMPs to induce necroptosis by binding to pattern recognition receptors once they are released to the cell extracellular space (32). The opening voltage-dependent anion channels (VDAC) promoted oxidative stress and mitochondrial dysfunction to influence the processes of necroptosis (33). *JMJD7-PLA2G4B* significantly increased starvation-induced cell death in head and neck squamous cell carcinoma (34). However, Petersen et al. elucidated that *TRAF2* can influence the association between RIPK3 and MLKL to inhibit necroptosis (35). Moreover, the inhibitory role of *DAPK1* and *RBCK1* in necroptosis has been discovered (36, 37). In summary, five of the abovementioned genes (*UCHL1*, *TRADD*, *H2AC6*, *VDAC3*, *JMJD7-PLA2G4B*) promote necroptosis and necroptosis-independent cell death, while the remaining three genes (*TFAF2*, *DAPK1*, and *RBCK1*) have roles in protecting cells from necroptosis.

To better evaluate the effect of the eight necroptosis-related genes in the prognostic model in COAD, we evaluated the expression levels of the eight necroptosis-related genes between the low-risk group and the high-risk group. The results demonstrated that the expression levels of *TRADD*, *H2AC6*, *JMJD7-PLA2G4B*, *TRAF2*, *UCHL1*, *DAPK1*, and *RBCK1* are higher in the high-risk group than those in the low-risk group, suggesting that the high expression of these genes in COAD is associated with poor prognosis. Then, we evaluated the expression of these eight necroptosis-related genes in tissues. Most of the genes were expressed differently between cancer tissues and normal tissues that we collected in RNA levels. The lack of statistical significance in the case of *TRADD* may be related to the small sample size. Otherwise, the protein expression levels of *TRADD*, *H2AC6*, *VDAC3*, *JMJD7-PLA2G4B*, *TRAF2*, and *DAPK1* in colon cancer tissues and normal tissues in the HPA database were shown, which were consistent with the expression levels of these genes in RNA levels. However, the data on the protein expression level of *UCHL1* were not detected and *RBCK1* in the HPA database is lacking. In the future, the protein expression level of necroptosis-related genes needed to be explored further. Furthermore, we used TNFα to induce necroptosis of cells and NEC-1 as RIPK1 inhibitor to inhibit the process of necroptosis. The expression of *DAPK1* decreased in cancer cells treated with TNFα and increased in cells treated with NEC-1, showing that *DAPK1* may influence the progression of cancer by inhibiting necroptosis. A previous study has also shown the inhibitory role of *DAPK1* in necroptosis in HT-29 cells, since knockdown or knockout of *DAPK1* in such cells increased cancer cell sensitivity to necroptosis (36). These results suggested that these eight LASSO genes may be vital prognostic factors for patients with COAD and may influence the progression of cancer by influencing necroptosis in COAD.

In terms of immunity analysis in our study, different risk groups showed diverse TME scores and immune infiltration landscape, suggesting that necroptosis-related genes may play a vital role in the immunity of COAD. Some studies have demonstrated that TME could have an impact on carcinogenesis and metastasis (28, 38). As the most abundant stromal cells, fibroblasts secrete various cytokines and remodel the extracellular matrix to promote tumor proliferation and metastasis (39). In our study, in the high-risk group associated with poorer OS, stromal cells and immune cells were more abundant, which suggests that disorders of stromal cells and immune cells promote the progression of cancer. On the other hand, B cells, macrophages, and T helper cells were more abundant in the high-risk group. Previous studies have shown that necroptotic cancer cells release DAMPs, chemokines, cytokines, and cancer antigens, which create an inflammatory immune microenvironment that can either have antitumor or protumor effects (40, 41). On the one hand, macrophages and DCs can be activated by necroptosis-derived DAMPs and cytokines, which can present antigens. Antigens recruit other immune cells to infiltrate the tumor and kill the cancer cells (42, 43). On the other hand, necroptotic cancer cells can attract tumor-associated macrophages which can cause immune suppression. Cytokines released by necroptotic cancer cells can also promote angiogenesis, cancer proliferation, and metastasis, combined with the release of ROS, which facilitates genomic instability and further contributes toward tumor progression (25). Moreover, a previous study demonstrated that RIPK3 blockade protects against pancreatic ductal adenocarcinoma progression *via* promoting infiltration of T cells and B cells (25). Overall, these findings suggest that necroptosis-related genes may have a potential impact on the function of the TME and immune cells in COAD and may become the target of immunotherapy in the future.

This study has several limitations. First, as the data in our study were acquired from public databases and the study was retrospective, there were some data that we could not collect. Thus, more prospective data are necessary to verify the clinical utility of the model. Second, we found only one high-throughput sequencing dataset with survival time information and a sample size greater than 40 of COAD specimens—GSE152430. Given that the TNM stage of all samples in the GSE152430 dataset is stage II, it is not adequate for us to validate the effectiveness of our model. In the future, with the popularity of high-throughput sequencing, we can further verify the effectiveness of our model based on large sample bioinformatics. Finally, we explored the effects of necroptosis on colon cancer cells and the expression of eight key necroptosis-related genes in the progression of necroptosis, but our study lacked basic experimental studies on the effects of necroptosis on immune cells and functional experiments of the eight key necroptosis-related genes. Further basic biological experiments are needed to further explore the mechanisms of necroptosis in COAD and tumor immunity.

## Conclusion

In our study, we identified a novel prognostic risk score model based on eight prognostic necroptosis-related DEGs in COAD. Then, we constructed a nomogram model to predict the prognosis of COAD patients. The immune and enrichment analyses suggested that necroptosis may be a vital mechanism for the progression and immunity of COAD.

## Data availability statement

The original contributions presented in the study are included in the article/**Supplementary Material**. Further inquiries can be directed to the corresponding authors.

## Ethics statement

Studies involving human participants were reviewed and approved by the Ethics Committee of the First Affiliated Hospital of Anhui Medical University. The patients/participants provided their written informed consent to participate in this study.

## Author contributions

Conceptualization: YW, M-GL, AX, S-CY, and Z-JW. Methodology: YW, M-GL, LM, and Z-MC. Formal analysis: YW and M-GL. Data curation: AX, S-CY, and Z-JW. Visualization: LM and Z-MC. Validation: LM and Z-MC. Writing—original draft preparation: YW and M-GL. Writing—review and editing: AX, S-CY, and Z-JW. Supervision: S-CY and Z-JW. Funding acquisition: AX. and Z-JW. All authors have read and agreed to the published version of the manuscript.

## Funding

This study was supported by the Clinical Medicine Discipline Construction Project of Anhui Medical University (2020lcxk032) and the Anhui Natural Science Foundation Youth Project (2108085QH337).

## Acknowledgments

The authors would like to sincerely thank the Department of General Surgery, First Affiliated Hospital of Anhui Medical University and the Department of Immunology, School of Basic Medical Sciences, Anhui Medical University for their valuable help in our study. We thank LetPub (https://www.letpub.com) for its linguistic assistance during the preparation of this manuscript.

## Conflict of interest

The authors declare that the research was conducted in the absence of any commercial or financial relationships that could be construed as a potential conflict of interest.

## Publisher’s note

All claims expressed in this article are solely those of the authors and do not necessarily represent those of their affiliated organizations, or those of the publisher, the editors and the reviewers. Any product that may be evaluated in this article, or claim that may be made by its manufacturer, is not guaranteed or endorsed by the publisher.

## Supplementary material

Supplementary Data sheet S1 Necroptosis-related genes with a relevance score above 1 included in our study.

Supplementary Data sheet S2 Primers sequences used in qRT-PCR in our study.

Supplementary Data sheet S3 The detailed results of functional enrichment analysis.

Supplementary Table S1 Clinical characteristics of our own COAD patients.

Supplementary Image S1 The expression of classical executors of necroptosis (RIPK1, RIPK3, MLKL, TNF) between the high and low risk groups in training cohort **(A)** and validation cohort **(B)**.

## Abbreviations

COAD, colon adenocarcinoma; TME, tumor microenvironment; TCGA, The Cancer Genome Atlas; AJCC, American Joint Committee on Cancer; TPM, transcripts per kilobase million; GTEx, Genotype-Tissue Expression; GEO, Gene Expression Omnibus; OS, overall survival; DEGs, differentially expressed genes; FDR, false discovery rate; HPA, Human Protein Atlas; LASSO, least absolute shrinkage and selection operator; PFS, progression-free survival; DCA, decision curve analysis; ssGSEA, single-sample gene set enrichment; GO: Gene Ontology; BP, biological processes; CC, cellular components; MF, molecular functions; IHC, immunohistochemistry; DMEM, Dulbecco’s modified Eagle’s medium; FBS, fetal bovine serum; NEC-1, necrostatin-1; qRT-PCR, quantitative real-time polymerase chain reaction; DCs, dendritic cells; DAMPs, damage-associated molecular patterns; ROS, reactive oxygen species

## References

[1] Ferlay J Colombet M Soerjomataram I Mathers C Parkin D Piñeros M. Estimating the global cancer incidence and mortality in 2018: GLOBOCAN sources and methods. Int J Cancer (2019) 144(8):1941–53. doi: 10.1002/ijc.31937 30350310

[2] Schmoll H Van Cutsem E Stein A Valentini V Glimelius B Haustermans K. ESMO consensus guidelines for management of patients with colon and rectal cancer. A personalized approach to clinical decision making. Ann Oncol Off J Eur Soc Med Oncol (2012) 23(10):2479–516. doi: 10.1093/annonc/mds236 23012255

[3] Bruni D Angell HK Galon J. The immune contexture and immunoscore in cancer prognosis and therapeutic efficacy. Nat Rev Cancer (2020) 20(11):662–80. doi: 10.1038/s41568-020-0285-7 32753728

[4] Nagtegaal ID Quirke P Schmoll HJ. Has the new TNM classification for colorectal cancer improved care? Nat Rev Clin Oncol (2011) 9(2):119–23. doi: 10.1038/nrclinonc.2011.157 22009076

[5] Wong R. Apoptosis in cancer: from pathogenesis to treatment. J Exp Clin Cancer Res CR (2011) 30:87. doi: 10.1186/1756-9966-30-87 21943236 PMC3197541

[6] Karsch-Bluman A Feiglin A Arbib E Stern T Shoval H Schwob O. Tissue necrosis and its role in cancer progression. Oncogene (2019) 38(11):1920–35. doi: 10.1038/s41388-018-0555-y 30390074

[7] Tang R Xu J Zhang B Liu J Liang C Hua J. Ferroptosis, necroptosis, and pyroptosis in anticancer immunity. J Hematol Oncol (2020) 13(1):110. doi: 10.1186/s13045-020-00946-7 32778143 PMC7418434

[8] Choi M Price D Ryter S Choi A. Necroptosis: a crucial pathogenic mediator of human disease. JCI Insight (2019) 4(15):e128834. doi: 10.1172/jci.insight.128834 31391333 PMC6693822

[9] Gong Y Fan Z Luo G Yang C Huang Q Fan K. The role of necroptosis in cancer biology and therapy. Mol Cancer (2019) 18(1):100. doi: 10.1186/s12943-019-1029-8 31122251 PMC6532150

[10] Qin X Ma D Tan Y Wang H Cai Z. The role of necroptosis in cancer: A double-edged sword? Biochim Biophys Acta Rev Cancer (2019) 1871(2):259–66. doi: 10.1016/j.bbcan.2019.01.006 30716362

[11] Moriwaki K Bertin J Gough PJ Orlowski GM Chan FK. Differential roles of RIPK1 and RIPK3 in TNF-induced necroptosis and chemotherapeutic agent-induced cell death. Cell Death Dis (2015) 6(2):e1636. doi: 10.1038/cddis.2015.16 25675296 PMC4669795

[12] Liu ZY Zheng M Li YM Fan XY Wang JC Li ZC. RIP3 promotes colitis-associated colorectal cancer by controlling tumor cell proliferation and CXCL1-induced immune suppression. Theranostics (2019) 9(12):3659–73. doi: 10.7150/thno.32126 PMC6587173 31281505

[13] Stelzer G Rosen N Plaschkes I Zimmerman S Twik M Fishilevich S. The GeneCards suite: From gene data mining to disease genome sequence analyses. Curr Protoc Bioinf (2016) 54:1.30.1–1.3. doi: 10.1002/cpbi.5 27322403

[14] Zhou Y Zhou B Pache L Chang M Khodabakhshi A Tanaseichuk O. Metascape provides a biologist-oriented resource for the analysis of systems-level datasets. Nat Commun (2019) 10(1):1523. doi: 10.1038/s41467-019-09234-6 30944313 PMC6447622

[15] Ju C Wyss R Franklin J Schneeweiss S Häggström J van der Laan M. Collaborative-controlled LASSO for constructing propensity score-based estimators in high-dimensional data. Stat Methods Med Res (2019) 28(4):1044–63. doi: 10.1177/0962280217744588 PMC6039292 29226777

[16] Wright M.N. Dankowski T. Ziegler A. Unbiased split variable selection for random survival forests using maximally selected rank statistics. Stat Med. (2017) 36(8):1272–1284. doi: 10.1002/sim.7212.12 28088842

[17] Iasonos A Schrag D Raj G Panageas K. How to build and interpret a nomogram for cancer prognosis. J Clin Oncol Off J Am Soc Clin Oncol (2008) 26(8):1364–70. doi: 10.1200/JCO.2007.12.9791 18323559

[18] Fitzgerald M Saville B Lewis R. Decision curve analysis. JAMA (2015) 313(4):409–10. doi: 10.1001/jama.2015.37 25626037

[19] Yoshihara K Shahmoradgoli M Martínez E Vegesna R Kim H Torres-Garcia W. Inferring tumour purity and stromal and immune cell admixture from expression data. Nat Commun (2013) 4:2612. doi: 10.1038/ncomms3612 24113773 PMC3826632

[20] Hänzelmann S Castelo R Guinney J. GSVA: Gene set variation analysis for microarray and RNA-seq data. BMC Bioinf (2013) 14:7. doi: 10.1186/1471-2105-14-7 PMC3618321 23323831

[21] Carbon P. D. Thomas L.P. Albou D. P. Hill P. Gaudet K. Van K. The gene ontology resource: 20 years and still GOing strong. Nucleic Acids Res (2019) 47:D330–D8. doi: 10.1093/nar/gky1055 PMC6323945 30395331

[22] Feng X Song Q Yu A Tang H Peng Z Wang X. Receptor-interacting protein kinase 3 is a predictor of survival and plays a tumor suppressive role in colorectal cancer. Neoplasma (2015) 62(4):592–601. doi: 10.4149/neo_2015_071 25997957

[23] Koo GB Morgan MJ Lee DG Kim WJ Yoon JH Koo JS. Methylation-dependent loss of RIP3 expression in cancer represses programmed necrosis in response to chemotherapeutics. Cell Res (2015) 25(6):707–25. doi: 10.1038/cr.2015.56 PMC4456623 25952668

[24] Strilic B Yang L Albarrán-Juárez J Wachsmuth L Han K Müller U. Tumour-cell-induced endothelial cell necroptosis *via* death receptor 6 promotes metastasis. Nature (2016) 536(7615):215–8. doi: 10.1038/nature19076 27487218

[25] Seifert L Werba G Tiwari S Giao Ly N Alothman S Alqunaibit D. The necrosome promotes pancreatic oncogenesis *via* CXCL1 and mincle-induced immune suppression. Nature (2016) 532(7598):245–9. doi: 10.1038/nature17403 PMC4833566 27049944

[26] Moriwaki K Balaji S McQuade T Malhotra N Kang J Chan F. The necroptosis adaptor RIPK3 promotes injury-induced cytokine expression and tissue repair. Immunity (2014) 41(4):567–78. doi: 10.1016/j.immuni.2014.09.016 PMC4220270 25367573

[27] Pasparakis M Vandenabeele P. Necroptosis and its role in inflammation. Nature (2015) 517(7534):311–20. doi: 10.1038/nature14191 25592536

[28] Singh R Mishra M Aggarwal H. Inflammation, immunity, and cancer. Mediators Inflammation (2017) 2017:6027305. doi: 10.1155/2017/6027305 PMC5695028 29234189

[29] Zeng F Chen X Cui W Wen W Lu F Sun X. RIPK1 binds MCU to mediate induction of mitochondrial Ca(2+) uptake and promotes colorectal oncogenesis. Cancer Res (2018) 78(11):2876–85. doi: 10.1158/0008-5472.CAN-17-3082 29531160

[30] Xu Y Gao H Hu Y Fang Y Qi C Huang J. High glucose-induced apoptosis and necroptosis in podocytes is regulated by UCHL1 *via* RIPK1/RIPK3 pathway. Exp Cell Res (2019) 382(2):111463. doi: 10.1016/j.yexcr.2019.06.008 31247189

[31] Wang L Chang X Feng J Yu J Chen G. TRADD mediates RIPK1-independent necroptosis induced by tumor necrosis factor. Front Cell Dev Biol (2019) 7:393. doi: 10.3389/fcell.2019.00393 32039207 PMC6987388

[32] Denning NL Aziz M Gurien SD Wang P. DAMPs and NETs in sepsis. Front Immunol (2019) 10:2536. doi: 10.3389/fimmu.2019.02536 31736963 PMC6831555

[33] Heslop KA Rovini A Hunt EG Fang D Morris ME Christie CF. JNK activation and translocation to mitochondria mediates mitochondrial dysfunction and cell death induced by VDAC opening and sorafenib in hepatocarcinoma cells. Biochem Pharmacol (2020) 171:113728. doi: 10.1016/j.bcp.2019.113728 31759978 PMC7309270

[34] Cheng Y Wang Y Li J Chang I Wang CY. A novel read-through transcript JMJD7-PLA2G4B regulates head and neck squamous cell carcinoma cell proliferation and survival. Oncotarget (2017) 8(2):1972–82. doi: 10.18632/oncotarget.14081 PMC5341748 28030848

[35] Petersen SL Chen TT Lawrence DA Marsters SA Gonzalvez F Ashkenazi A. TRAF2 is a biologically important necroptosis suppressor. Cell Death Diff (2015) 22(11):1846–57. doi: 10.1038/cdd.2015.35 PMC4648330 25882049

[36] Wu YH Chou TF Young L Hsieh FY Pan HY Mo ST. Tumor suppressor death-associated protein kinase 1 inhibits necroptosis by p38 MAPK activation. Cell Death Dis (2020) 11(5):305. doi: 10.1038/s41419-020-2534-9 32366830 PMC7198492

[37] Taraborrelli L Peltzer N Montinaro A Kupka S Rieser E Hartwig T. LUBAC prevents lethal dermatitis by inhibiting cell death induced by TNF, TRAIL and CD95L. Nat Commun (2018) 9(1):3910. doi: 10.1038/s41467-018-06155-8 30254289 PMC6156229

[38] Thompson E Zahurak M Murphy A Cornish T Cuka N Abdelfatah E. Patterns of PD-L1 expression and CD8 T cell infiltration in gastric adenocarcinomas and associated immune stroma. Gut (2017) 66(5):794–801. doi: 10.1136/gutjnl-2015-310839 26801886 PMC4958028

[39] Joshi R Kanugula S Sudhir S Pereira M Jain S Aghi M. The role of cancer-associated fibroblasts in tumor progression. Cancers (Basel) (2021) 13(6):1399. doi: 10.3390/cancers13061399 33808627 PMC8003545

[40] McCracken M Cha A Weissman I. Molecular pathways: Activating T cells after cancer cell phagocytosis from blockade of CD47 "Don't eat me" signals. Clin Cancer Res Off J Am Assoc Cancer Res (2015) 21(16):3597–601. doi: 10.1158/1078-0432.CCR-14-2520 PMC4621226 26116271

[41] Nagarsheth N Wicha M Zou W. Chemokines in the cancer microenvironment and their relevance in cancer immunotherapy. Nat Rev Immunol (2017) 17(9):559–72. doi: 10.1038/nri.2017.49 PMC5731833 28555670

[42] Koks C Garg A Ehrhardt M Riva M Vandenberk L Boon L. Newcastle Disease virotherapy induces long-term survival and tumor-specific immune memory in orthotopic glioma through the induction of immunogenic cell death. Int J Cancer (2015) 136(5):E313–25. doi: 10.1002/ijc.29202 25208916

[43] Aaes T Kaczmarek A Delvaeye T De Craene B De Koker S Heyndrickx L. Vaccination with necroptotic cancer cells induces efficient anti-tumor immunity. Cell Rep (2016) 15(2):274–87. doi: 10.1016/j.celrep.2016.03.037 27050509

