# Supplementary material for: Identification of necroptosis-related genes for predicting prognosis and exploring immune infiltration landscape in colon adenocarcinoma
Source: Front Oncol. 2022 Nov 24;12:941156. doi: 10.3389/fonc.2022.941156 (PMC9731216; doi:10.3389/fonc.2022.941156)
Supplement: Supplementary Table S1 — Clinical characteristics of our own COAD patients. [file Table_1.docx]

**Additional file 5:** Clinical characteristics of our own COAD patients

| **Variables** | **Patients** |
| --- | --- |
| **Age** |  |
| ≤65 | 6 (37.5%) |
| ＞65 | 10 (62.5%) |
| **Gender** |  |
| Female | 4 (25.0%) |
| Male | 12 (75.0%) |
| **T stage** |  |
| T1-T2 | 1 (6.25%) |
| T3 | 15 (93.75%) |
| T4 | 0 (0%) |
| **N stage** |  |
| N0 | 8 (50.0%) |
| N1 | 5 (31.25%) |
| N2 | 3 (18.75%) |
| **M stage** |  |
| M0 | 16 (100.0%) |
| M1 | 0 (0.0%) |
| **Site of tumor** |  |
| ascending colon  transverse colon  descending colon  sigmoid colon | 8 (50.0%)  1 (6.25%)  2 (12.5%)  5 (31.25%) |
| **Differentiated** |  |
| moderately | 15 (93.75%) |
| poorly | 1 (6.25%) |
