# Supplementary figures and images for: Identification of necroptosis-related genes for predicting prognosis and exploring immune infiltration landscape in colon adenocarcinoma
Source: Front Oncol. 2022 Nov 24;12:941156. doi: 10.3389/fonc.2022.941156 (PMC9731216; doi:10.3389/fonc.2022.941156)

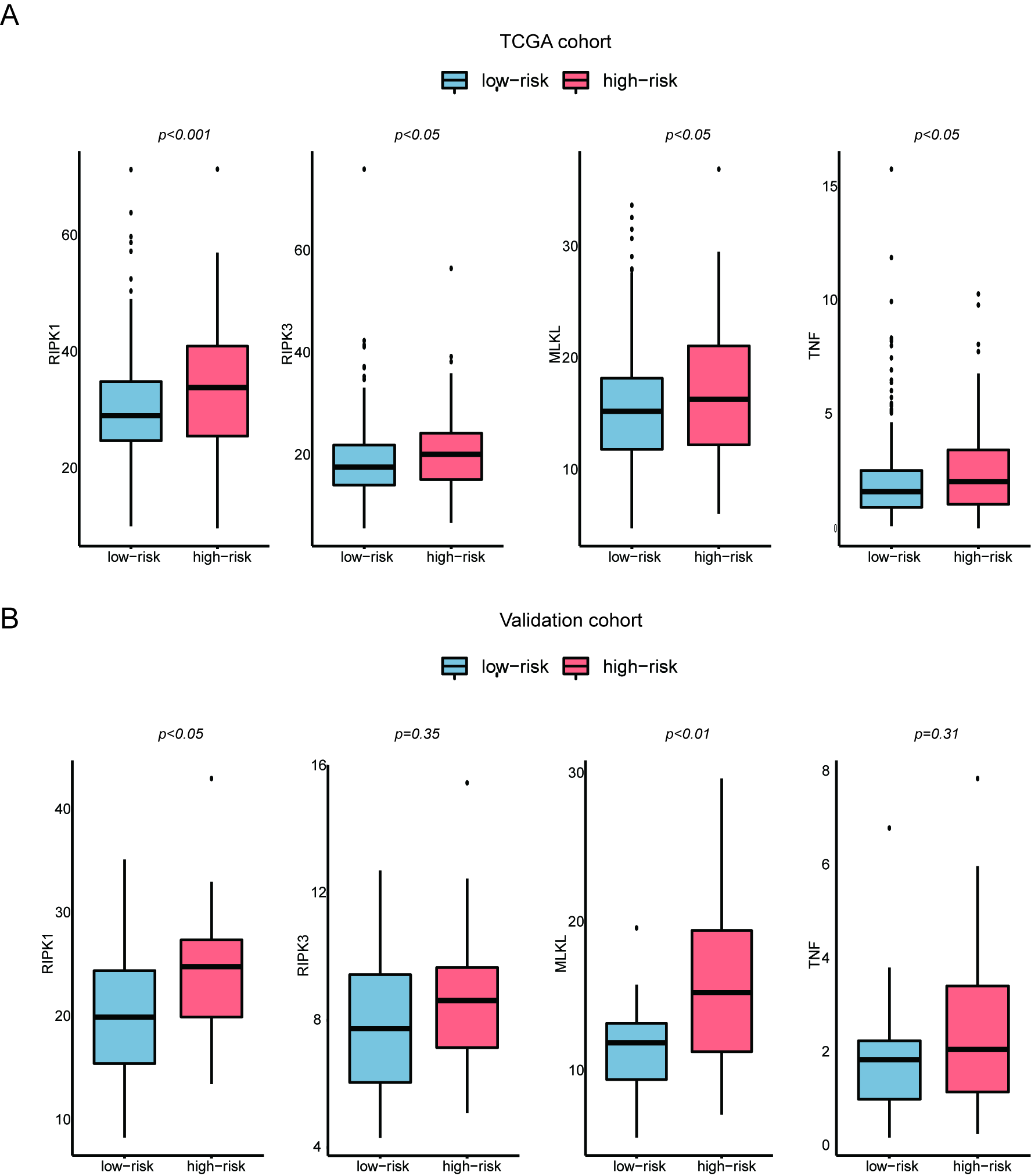

Supplement: Supplementary Image S1 — The expression of classical executors of necroptosis (RIPK1, RIPK3, MLKL, TNF) between the high and low risk groups in training cohort (A) and validation cohort (B). [file Image_1.tif]
